# Attitudes and Experiences of Clinicians After Mandated Implementation of Open Notes by the 21st Century Cures Act: Survey Study

**DOI:** 10.2196/42021

**Published:** 2023-02-28

**Authors:** Sophia M Leonard, Rosalee Zackula, Jonathan Wilcher

**Affiliations:** 1 Kansas City Campus The University of Kansas School of Medicine Kansas City, KS United States; 2 Wichita Campus Office of Research The University of Kansas School of Medicine Wichita, KS United States; 3 Kansas City Campus Department of Emergency Medicine The University of Kansas Health System Kansas City, KS United States

**Keywords:** 21st Century Cures Act, Final Rule, shared notes, open notes, OpenNotes, health policy, clinician opinion, mobile phone

## Abstract

**Background:**

On December 13, 2016, the US Congress enacted the 21st Century Cures Act (hereafter the Cures Act), which contained the Final Rule mandate that took effect on April 5, 2021. Since then, health systems have been required to provide patients digital access to their eHealth information “without delay” and without charge.

**Objective:**

This study aimed to assess clinicians’ initial experiences with, and attitudes toward, sharing visit notes with patients after being mandated to do so by the Cures Act and to determine clinician preferences regarding instant record release.

**Methods:**

This cross-sectional survey study was conducted between June 10, 2021, and August 15, 2021, at the University of Kansas Health System, a large academic medical center in Kansas City, Kansas, United States. Participants included clinicians currently employed by the health system, including resident and attending physicians, physician assistants, nurse practitioners, and critical care and emergency medicine registered nurses.

**Results:**

A total of 1574 attending physicians, physician assistants, and nurse practitioners, as well as 506 critical care and emergency medicine nurses, were sent invitations; 538 (34.18%) and 72 (14.2%), respectively, responded. Of 609 resident physicians, 4 (response rate not applicable because it was unknown how many residents viewed the website while the link was available) responded. The majority of respondents were attending physicians (402/614, 65.5%) and within the department of internal medicine (160/614, 26.1%). Most agreed that sharing visit notes was a good idea (355/613, 57.9%) and that it is important to speak with the patients before they accessed their records (431/613, 70.3%). Those who agreed that sharing visit notes is a good idea tended to view the practice as a useful tool for engaging patients (“Agree”: 139/355, 39.2%; “Somewhat agree”: 161/355, 45.4%; *P<*.001) and experience no change in the clinical value of their notes for other clinicians (326/355, 91.8%; *P<*.001). Those who disagreed (or were neutral) tended not to encourage patients to read their notes (235/258, 91.1%; *P<*.001) and were more likely to experience a change in their charting practice (168/257, 65.4%; *P<*.001) and increased time charting (99/258, 38.4%; *P<*.001).

**Conclusions:**

The findings of this study may be generalizable to institutions similar to the University of Kansas Health System, and the clinician testimonies gathered in this study may provide valuable insight into the initial opinions and experiences of clinicians at these institutions. In addition, these clinician experiences collected early in the transition period may be used to guide future health policy implementation and to understand how best to prepare clinicians for these changes in practice.

## Introduction

### Background

After the Affordable Care Act was enacted in 2012, patient-centered care became a central goal of the US healthcare system [1]. Reflected in this goal is the desire to increase transparency and promote patient involvement in their health care. In 2010, a pilot study was conducted by Beth Israel Deaconess Medical Center, in which primary care physicians were recruited to participate in a practice called “open notes” in which they would provide patient volunteers with their clinical notes. For context, the clinical note (aka visit note) is where information about the patient encounter is documented and often includes information such as the clinician’s impression of the patient, physical examination findings, and differential diagnoses, as well as the patient’s medical, surgical, and social history. It is often used as a tool for communication among members of the patient’s healthcare team, allowing for continuity of care. After a year of sharing notes, the physicians and their patients were surveyed, revealing beneficial patient effects and modest changes to physician practice [2]. Subsequent research at the participating institutions and elsewhere continued to report patient benefits of note sharing [3-9], eventually leading to an international movement promoting the practice, commonly known as OpenNotes.

On December 13, 2016, the US Congress enacted the 21st Century Cures Act (hereafter the Cures Act) [10], which contained the Final Rule mandate that went into effect on April 5, 2021. Since then, health systems have been required to provide patients digital access to their eHealth information “without delay” and without charge. Some of the data elements now readily shared with patients via web-based patient portals or smartphone apps are history and physicals; narratives from imaging, laboratory, and pathology reports; and notes from consultations, procedures, and progress, along with discharge summaries. Although there are some exceptions to sharing clinical notes, rigorous conditions must be met to qualify for these exceptions [10]. Of note, many have considered the Cures Act Final Rule to be the *OpenNotes Rule* [11]. However, this is incorrect. Although implementation of the Final Rule mandate now requires clinicians to share notes with patients, the Cures Act is a health policy with the goal of providing patients with free, uninhibited digital access to their medical records. It has additional implications beyond sharing notes, such as the instant release of records.

Research on the effects of sharing visit notes has primarily focused on the patient experience, whereas studies assessing clinician experience have been limited. These are confined to a few medical specialties, anticipatory studies, and studies conducted within health systems that voluntarily began sharing clinical notes. Although physicians anticipated improved patient engagement from note sharing, conflicting attitudes were also reported [12-14]; for example, surveys with clinician specialists revealed concerns about the impact on documentation, specifically, modifying the tone and including fewer details [15-17]. In addition, a fear of potentially harming the physician-patient relationship [12,13] or causing greater patient worry [12,14] was reported. Two interdisciplinary studies examining clinician attitudes toward sharing visit notes revealed that most of the clinicians considered the practice to be a good idea [18,19]. However, both studies were conducted within institutions that willingly adopted the practice and a considerable amount of time had elapsed after starting to share notes. Voluntary adoption of the practice suggests institutional approval of the practice and control over factors such as which clinicians and patients initially participated and the rate at which the practice was implemented institution wide. In addition, these clinician study participants did not have to contend with the other Cures Act requirements of information blocking and the instantaneous release of records. These study setting characteristics may have resulted in a bias toward a more favorable clinician opinion of open notes. Furthermore, most of the studies conducted before the Cures Act only examined the experience of physicians and not additional disciplines such as advanced practice providers and registered nurses.

### Objectives

To better understand the clinician experience in the wake of the Cures Act and to identify areas for improvement and the potential need for clinician intervention, a survey was conducted at a large Midwest academic medical center soon after the implementation of the Final Rule mandate. The primary objective of this study was to assess clinicians’ initial experiences with, and attitudes toward, sharing visit notes with patients after being mandated to do so by the Cures Act. The secondary objective was to determine clinician preferences regarding instant record release.

## Methods

### Setting

The survey was conducted with a cross-section of healthcare professionals at the University of Kansas Health System in Kansas City, Kansas, United States, whose patients have had access to health records via MyChart, the institutional web-based patient portal, since the implementation of the Cures Act. All hospital specialties and major professional disciplines were included to provide a comprehensive view of the initial impact of the new practice. Data were collected via questionnaire from June 10, 2021, to August 15, 2021. This manuscript was prepared in adherence to the Checklist for Reporting of Survey Studies guidelines [20].

### Ethical Considerations

In April 2021, the proposal was submitted to the University of Kansas Medical Center institutional review board, and it determined that no approval was required because only deidentified data were used.

### Participants

**Figure 1:**
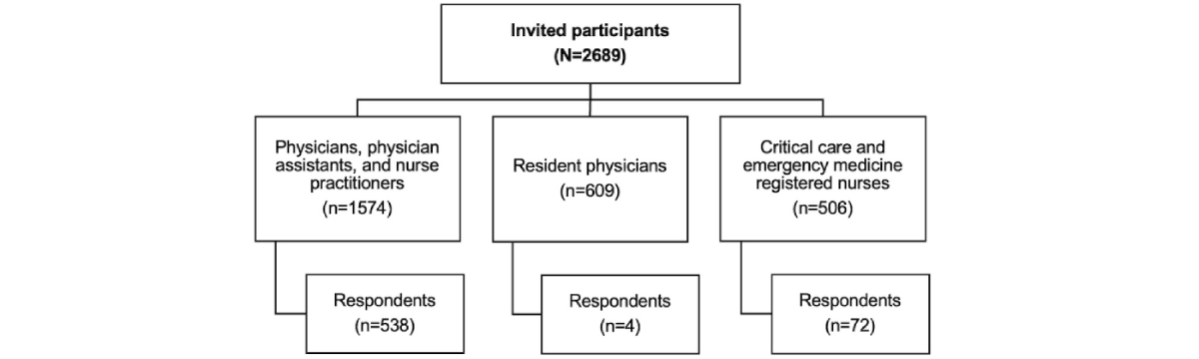
Study flowchart.

Participants were clinicians currently employed by the health system and included residents, attending physicians, advanced practice providers (ie, physician assistants and nurse practitioners), along with critical care and emergency medicine registered nurses. At the University of Kansas Health System, critical care nurses work within the emergency room and intensive care units (ICUs) such as the medical ICU, cardiac ICU, neuroscience ICU, otolaryngology and head and neck ICU, and all surgical ICUs. Figure 1 shows the study flowchart.

### Constructing the Questionnaire

The questionnaire consisted of 11 items developed from a previously published instrument assessing the views and experiences of clinicians after voluntarily sharing visit notes with patients [2,18]. Additional questions were devised to assess clinician preferences regarding instant record release and potential changes in charting. Clinician demographics, including professional discipline and specialty, were also collected. To protect participant anonymity, additional participant information, such as age and sex, was not recorded. The questionnaire is available in Multimedia Appendix 1.

### Recruitment

A request for participants was distributed to attending physicians and advanced practice providers through their institutional email addresses via REDCap (Research Electronic Data Capture; Vanderbilt University) [21,22], a secure web-based data management application. Each invitation included a unique link to the survey that was deactivated after a response was entered, thus controlling for multiple participation. Because of health system and graduate medical education policies, mass distribution of the survey to resident physicians and registered nurses was prohibited, and participation required approval by the resident program administrator and directors of nursing for each specialty. In the end, the survey was distributed to resident physicians by the university program administrator, who posted a link to the questionnaire on the institutional resident website for voluntary participation. The director of nursing for critical care services agreed to participate and facilitated the survey by emailing links to the institutional email addresses of critical care and emergency medicine nurses.

### Statistical Analysis

The primary outcome for this study was a dichotomous response, *Favored* versus *Not favored*, to the statement “Making visit notes available to patients is a good idea.” Originally, responses were from a 5-point Likert-scale item with selections for “Agree,” “Somewhat agree,” “Neutral,” “Somewhat disagree,” and “Disagree.” Descriptive statistics included counts and percentages from the questionnaire. Chi-square tests of independence were conducted among questionnaire responses using SPSS software (version 26.0; IBM Corp) to evaluate associations with the dichotomized primary outcome. Polychoric correlations were computed using the R Hector package in SPSS to measure the strength of linear association among responses. Values of |0.2 to 0.4| were defined as weak associations, |0.4 to 0.6| as moderate, and |0.6 to 0.8| as strong. Missing responses were negligible. Of the 614 participants, only 5 (0.8%) had incomplete responses; thus, no imputations were performed. Qualitative analysis of participant responses to open-ended portions of the survey was conducted via manual coding using grounded theory to identify common themes and sentiments.

## Results

### Participants

A total of 1574 survey invitations, with up to 5 reminder emails (5833 in total), were sent to attending physicians and advanced practice providers via REDCap. An additional 506 critical care and emergency medicine registered nurses were provided a link to the survey instrument by the director of nursing. A total of 609 residents had access to the survey via the institutional website. Overall, 614 responses were recorded, 5 of which were incomplete. The response rate for the attending physicians (n=402) and advanced practice providers (n=136) was 34.18% (538/1574). Respondents also included registered nurses (72/506, 14.2%), along with resident physicians (4/609; response rate not applicable because it was unknown how many residents viewed the website while the link was available). Table 1 summarizes item responses and shows the number of missing responses. It should be noted that the department with the highest participation was internal medicine with a response rate of 26.1% (160/614). The majority of respondents (355/613, 58%) seemed to support the practice of open notes, with 43.1% (153/355) agreeing and 56.9% (202/355) somewhat agreeing that making visit notes available was a good idea. More than half of the participants (323/612, 52.8%) also reported that open notes had changed how they chart. The bulk of the clinicians (431/613, 70.3%) also agreed that it was important to speak with patients before they accessed their records. Furthermore, when asked whether open notes were a useful tool for engaging patients in their care, 55.5% (340/613) agreed or somewhat agreed, providing additional support of the practice.

**Table 1:** Respondent summary (N=614).

Survey questions			Values, n (%)
**What department do you work in? (missing responses: 0)**			
	Anesthesiology	44 (7.2)	
	Cardiovascular medicine	49 (8)	
	Emergency medicine	46 (7.5)	
	Family medicine	31 (5)	
	General surgery	27 (4.4)	
	Internal medicine	160 (26.1)	
	Neurology	31 (5)	
	Neurosurgery	17 (2.8)	
	Obstetrics and gynecology	17 (2.8)	
	Ophthalmology	7 (1.1)	
	Orthopedic surgery	12 (1.9)	
	Otolaryngology and head and neck surgery	18 (2.9)	
	Pathology and laboratory medicine	2 (0.3)	
	Pediatrics	34 (5.5)	
	Plastic surgery	9 (1.5)	
	Psychiatry and behavioral sciences	19 (3.1)	
	Radiation oncology	6 (1)	
	Radiology	8 (1.3)	
	Rehabilitation medicine	10 (1.6)	
	Urological surgery	12 (2)	
	Other	55 (9)	
**What is your position within the department? (missing responses: 0)**			
	Attending physician	402 (65.5)	
	Resident physician	4 (0.7)	
	Advanced practice provider	136 (22.1)	
	Registered nurse	72 (11.7)	
**How much do you agree that making visit notes available is a good idea? (missing responses: 1)**			
	Agree	153 (25)	
	Somewhat agree	202 (33)	
	Neutral	65 (10.6)	
	Somewhat disagree	128 (20.9)	
	Disagree	65 (10.6)	
**How much do you agree that open notes are a useful tool for engaging patients in their care? (missing responses: 1)**			
	Agree	142 (23.2)	
	Somewhat agree	198 (32.3)	
	Neutral	102 (16.6)	
	Somewhat disagree	113 (18.4)	
	Disagree	58 (9.4)	
**Is it important to you that you speak to your patients about certain records prior to them accessing the records?^a^ (missing responses: 1)**			
	No	180 (29.4)	
	Yes	431 (70.3)	
**Because of open notes do you spend... (missing responses: 0)**			
	More time writing notes	184 (30)	
	No change	405 (66.1)	
	Less time writing notes	24 (3.9)	
**Do you believe open notes has changed the way you chart? (missing responses: 2)**			
	No	289 (47.2)	
	Yes	323 (52.8)	
**Has open notes affected the clinical value of your notes for other clinicians? (missing responses: 1)**			
	More valuable	7 (1.1)	
	No change	520 (84.8)	
	Less valuable	86 (14)	
**Do you encourage your patients to read their notes? (missing responses: 2)**			
	Yes	142 (23.2)	
	No	470 (76.8)	

^a^If the participant selected “yes” to this question, a subsequent question was displayed, inquiring about which kinds of records (eg, laboratory test results and imaging studies) they would like patients to have delayed access to.

### Participant Responses by Position

Next, clinician responses by departmental position were explored; the results are shown in Table 2 (on the basis of the sparse number of resident physician participants, response data from this population are not reported in Table 2). In total, 32.8% (132/402) of the attending physicians, along with 19.9% (27/136) of the practitioners, belonged to internal medicine, and 28% (20/72) of the nurses were from emergency medicine. In support of open notes, 59.9% (241/402) of the attendings said that making visit notes available was a good idea, compared with 59.3% (80/135) of the practitioners and 44% (32/72) of the nurses. The proportions that said shared notes were a useful tool for engaging patients were similar: 57.4% (231/402) of the attendings, 59.3% (80/135) of the practitioners, and 38% (27/72) of the nurses. Therefore, registered nurses tended to have a negative or neutral opinion of open notes.

The majority of clinicians, regardless of position, reported “Yes,” it is important to speak with patients before they access their records. Responses included 71.6% (288/402) of the attending physicians, 71.1% (96/135) of the advanced practice providers, and 65% (47/72) of the registered nurses. The participants who responded “Yes” to this question (n=431) were then asked what kinds of records they would like patients to have delayed access to. A majority of respondents chose results from laboratory tests (339/431, 78.6%) along with radiology results (375/431, 87%).

A particular emphasis was placed on pathology results pertaining to cancer diagnoses (166/431, 38.5%). One respondent voiced their concern in a text box, stating the following:

I have never had patients angrier about lack of communication than when a patient saw a result positive for cancer prior to me calling them. The result was released instantly, and I had NO CHANCE to call the patient prior to their notification. This has impacted my practice, as now I tell patients they will probably see results before I do.

Participant 1

Another commented as follows:

We have had two patients find out they had cancer on their birthdays, for example. Never mind all the anxiety over benign findings that, with a Google search to the uneducated, can result in the conclusion of worst possible scenario. It is a really stupid idea this was done.

Participant 2

A few of the respondents (13/168, 8%) expanded on their preference for delayed records and described how the instantaneous release of reports has directly affected their well-being. One spoke about an increased sense of burnout, stating, “Time required in phone calls and EMR messaging is unmeasured, unmonitored and is yet another form of abuse of clinicians in [unreimbursed] time that helps make the concept of work life balance a hypocritical joke being foisted on clinicians victimized by government, employers, and patients themselves.” Another clinician discussed how the practice leads to patient distrust of physicians, stating as follows:

Notes are written for doctor-to-doctor communication. I get so many comments about how doctors lie because they did not do this or they didn’t tell me they were looking for XYZ, so now I can’t trust them, and I won’t go back to them.

Participant 3

Most of the participants (405/613, 66.1%) reported no change in time spent charting, although the results varied by position: 63.7% (256/402) of the attending physicians, 74.1% (100/135) of the practitioners, and 64% (46/72) of the registered nurses. Of the 323 respondents who reported changing the way they chart, the majority were attendings (221/323, 68.4%): 34.6% (139/402) took more time to write notes; 83.3% (184/221) changed how they documented sensitive clinical, mental health, or social information; 78.7% (174/221) changed the use of language that could be perceived as critical to the patients; and 65.2% (144/221) reported that they stopped using terms such as “noncompliant,” “patient refuses,” and “patient denies.” Using medical jargon or abbreviations was the least likely change reported by attending physicians (74/221, 33.5%). Those who selected “other” changes in charting practices were prompted to provide details in a textbox. Among the responses, a major theme that surfaced was that medical records are now written more with the patient in mind than other clinicians. One clinician commented, “Frankly, I think it makes me chart with the intention of the patient reading the chart rather than another provider reading the chart and I think, depending on the situation, this actually compromises the quality of the note.” Another stated, “Documentation is less accurate as using correct medical terms is confusing to patients—you almost need two notes, one in a language understandable for the patient and another note to accurately convey medical information to other providers.” It was also noted that some clinicians now avoid documenting specific differential diagnoses to prevent patient anxiety. In addition, some clinicians reported changes in charting owing to concern regarding the privacy of documented sensitive information, particularly as it pertains to adolescents and those experiencing domestic abuse. One respondent noted as follows:

[I] can no longer comment on sensitive maternal health issues/concerns for domestic violence relevant to the overall plan for the child in the chart as [the] partner would also have access to the child’s notes. This makes interdisciplinary communication prone to missing vital information.

Participant 4

Additional clinician testimony is available in Multimedia Appendix 2.

Although most of the clinicians (323/612, 52.8%) reported a change in charting, a large majority also said that this has not affected the clinical value of their notes for other clinicians (attending physicians: 332/402, 82.6%; advanced practice providers: 121/135, 89.6%; and registered nurses, 64/72, 89%). Not shown in Table 2 are several clinicians who commented that their notes are now less direct, less efficient, less accurate, objective, and technical. Moreover, they stated that now their notes also contain clinically irrelevant information.

**Table 2:** Clinician responses by departmental position (N=610).

Questions and responses				Attendings (n=402), n (%)		Practitioners (n=136), n (%)		Nurses (n=72), n (%)
**What department of the University of Kansas Hospital do you work in?**								
	Anesthesiology		27 (6.7)		17 (12.5)		0 (0)	
	Cardiovascular medicine		17 (4.2)		17 (12.5)		15 (20.8)	
	Emergency medicine		19 (4.7)		5 (3.7)		20 (27.8)	
	Family medicine		25 (6.2)		6 (4.4)		0 (0)	
	General surgery		16 (4)		5 (3.7)		6 (8.3)	
	Internal medicine		132 (32.8)		27 (19.9)		0 (0)	
	Neurology		22 (5.5)		6 (4.4)		3 (4.2)	
	Neurosurgery		9 (2.2)		0 (0)		8 (11.1)	
	Obstetrics and gynecology		14 (3.5)		3 (2.2)		0 (0)	
	Ophthalmology		7 (1.7)		0 (0)		0 (0)	
	Orthopedic surgery		10 (2.5)		2 (1.5)		0 (0)	
	Otolaryngology and head and neck surgery		13 (3.2)		4 (2.9)		1 (1.4)	
	Pathology and laboratory medicine		2 (0.5)		0 (0)		0 (0)	
	Pediatrics		25 (6.2)		9 (6.6)		0 (0)	
	Plastic surgery		7 (1.7)		0 (0)		2 (2.8)	
	Psychiatry and behavioral sciences		17 (4.2)		1 (0.7)		0 (0)	
	Radiation oncology		5 (1.2)		1 (0.7)		0 (0)	
	Radiology		7 (1.7)		1 (0.7)		0 (0)	
	Rehabilitation medicine		5 (1.2)		0 (0)		5 (6.9)	
	Urological surgery		9 (2.2)		3 (2.2)		0 (0)	
	Other		14 (3.5)		29 (21.3)		12 (16.7)	
**How much do you agree that making visit notes available is a good idea?**								
	Agree		109 (27.1)		34 (25.2)		9 (12.5)	
	Somewhat agree		132 (32.8)		46 (34.1)		23 (31.9)	
	Neutral		38 (9.5)		19 (14.1)		8 (11.1)	
	Somewhat disagree		84 (20.9)		23 (17)		19 (26.4)	
	Disagree		39 (9.7)		13 (9.6)		13 (18.1)	
**How much do you agree that open notes are a useful tool for engaging patients in their care?**								
	Agree		97 (24.1)		31 (23)		13 (18.1)	
	Somewhat agree		134 (33.3)		49 (36.3)		14 (19.4)	
	Neutral		59 (14.7)		26 (19.3)		16 (22.2)	
	Somewhat disagree		79 (19.7)		18 (13.3)		16 (22.2)	
	Disagree		33 (8.2)		11 (8.1)		13 (18.1)	
**Do you encourage your patients to read their notes?**								
	Yes		98 (24.4)		29 (21.5)		14 (19.7)	
	No		304 (75.6)		106 (78.5)		57 (80.3)	
**Is it important to you that you speak to your patients about certain records prior to them accessing the records?**								
	Yes		288 (71.6)		96 (71.1)		47 (65.3)	
	No		114 (28.4)		39 (28.9)		25 (34.7)	
**Because of open notes do you spend...**								
	More time writing notes		139 (34.6)		33 (24.4)		11 (15.3)	
	No change		256 (63.7)		100 (74.1)		46 (63.9)	
	Less time writing notes		7 (1.7)		2 (1.5)		15 (20.8)	
**Do you believe open notes has changed the way you chart?**								
	No		180 (44.9)		75 (55.6)		34 (47.2)	
	Yes		221 (55.1)		60 (44.4)		38 (52.8)	
	**If answered “Yes,” choose all that apply:**							
		Use of language could be perceived as critical to the patient	174 (78.7)		46 (76.7)		20 (52.6)	
		How you document sensitive clinical, mental health, or social information	184 (83.3)		51 (85)		32 (84.2)	
		How you document patients’ perspectives, preferences, and concerns	136 (61.5)		42 (70)		25 (65.8)	
		Use of terms such as “noncompliant,” “patient refuses,” and “patient denies”	144 (65.2)		45 (75)		28 (73.7)	
		Use of medical jargon or abbreviations	74 (33.5)		22 (36.7)		15 (39.5)	
		Other	16 (7.2)		1 (1.7)		1 (2.6)	
**Has open notes affected the clinical value of your notes for other clinicians?**								
	More valuable		3 (0.7)		1 (0.7)		2 (2.8)	
	No change		332 (82.6)		121 (89.6)		64 (88.9)	
	Less valuable		67 (16.7)		13 (9.6)		6 (8.3)	

### Clinician Opinion of and Experience With Shared Notes

Survey responses were also explored by the dichotomized outcome *Favored* (355/613, 57.9%) versus *Not favored* (258/613, 42.1%) regarding making notes available to patients (Table 3). Those in the *Favored* category were significantly more likely to view shared notes as valuable tools for engaging patients (“Agree”: 139/355, 39.2%; “Somewhat agree”: 161/355, 45.4%; *P<*.001), with 91.8% (326/355; *P<*.001) reporting no change in the clinical value of their notes.

Conversely, those categorized as *Not favored* were more likely to report that they did not encourage patients to read their notes (235/258, 91.1%; *P<*.001) and that sharing notes changed the way they chart (168/257, 65.4%; *P<*.001). Of this population, 38.4% (99/258; *P<*.001) said that the practice increased time for writing notes, and 24.8% (64/258; *P<*.001) now considered their notes to be less valuable for other clinicians.

**Table 3:** Comparing responses based on clinician opinion regarding making visit notes available to patients (N=613).

			Making visit notes available to patients is a good idea				*P* value
			Not favored (n=258), n (%)		Favored (n=355), n (%)		
**Hospital department**							.66
	Anesthesiology	17 (6.6)		26 (7.3)			
	Cardiovascular medicine	22 (8.5)		27 (7.6)			
	Emergency medicine	21 (8.1)		25 (7)			
	Family medicine	13 (5)		18 (5.1)			
	General surgery	16 (6.2)		11 (3.1)			
	Internal medicine	70 (27.1)		90 (25.4)			
	Neurology	10 (3.9)		21 (5.9)			
	Neurosurgery	11 (4.3)		6 (1.7)			
	Obstetrics and gynecology	8 (3.1)		9 (2.5)			
	Ophthalmology	2 (0.8)		5 (1.4)			
	Orthopedic surgery	5 (1.9)		7 (2)			
	Otolaryngology and head and neck surgery	9 (3.5)		9 (2.5)			
	Pathology and laboratory medicine	1 (0.4)		1 (0.3)			
	Pediatrics	8 (3.1)		26 (7.3)			
	Plastic surgery	4 (1.6)		5 (1.4)			
	Psychiatry and behavioral sciences	7 (2.7)		12 (3.4)			
	Radiation oncology	3 (1.2)		3 (0.8)			
	Radiology	2 (0.8)		6 (1.7)			
	Rehabilitation medicine	4 (1.6)		6 (1.7)			
	Urological surgery	5 (1.9)		7 (2)			
	Other	20 (7.8)		35 (9.9)			
**Hospital position**							.09
	Attending physician	161 (62.4)		241 (67.9)			
	Resident physician	2 (0.8)		2 (0.6)			
	Advanced practice provider	55 (21.3)		80 (22.5)			
	Registered nurse	40 (15.5)		32 (9)			
**Open note is useful tool for engaging patient**							<.001
	Agree	3 (1.2)		139 (39.2)			
	Somewhat agree	37 (14.3)		161 (45.4)			
	Neutral	70 (27.1)		32 (9)			
	Somewhat disagree	92 (35.7)		21 (5.9)			
	Disagree	56 (21.7)		2 (0.6)			
**Open note charting time**							<.001
	More time writing notes	99 (38.4)		85 (23.9)			
	No change	142 (55)		263 (74.1)			
	Less time writing notes	17 (6.6)		7 (2)			
**Open note clinical value**							<.001
	More valuable	0 (0)		7 (2)			
	No change	194 (75.2)		326 (91.8)			
	Less valuable	64 (24.8)		22 (6.2)			
**I encourage patient to read notes**							<.001
	Yes	23 (8.9)		119 (33.6)			
	No	235 (91.1)		235 (66.4)			
**Important to speak with patient before they access their notes**							.79
	Yes	184 (71.3)		249 (70.1)			
	No	74 (28.7)		106 (29.9)			
**Changes the way I chart**							<.001
	Yes	168 (65.4)		155 (43.7)			
	No	89 (34.6)		200 (56.3)			

### Bivariate Correlations

To better understand the relationships among responses, polychoric correlations were conducted (Table 4). Three associations were rated as moderate (50%-69%) to strong (≥70%). Respondents who agreed that sharing visit notes is a good idea also tended to agree that it is a valuable tool for engaging patients (*r*=0.83). Of the 355 respondents who agreed or somewhat agreed, 300 (84.5%) believed that it is a valuable tool, whereas 55 (15.5%) did not. Of the 258 respondents who disagreed, somewhat disagreed, or were neutral regarding their opinion of open notes, 40 (15.5%) stated that they thought it is a valuable tool, whereas the remaining 218 (84.5%) disagreed or were neutral regarding their opinion.

Those who believe that sharing notes has changed how they chart also tended to experience increased charting time (*r*=–0.51). Of the 323 participants who reported a change in their charting, 169 (52.3%) said that they now spend more time charting; for 132 (40.9%), there was no difference; and 22 (6.8%) said that they spend less time. An additional notable relationship was demonstrated between change in charting and the effect that shared visit notes have had on clinician notes’ clinical value (*r*=0.54). Of those who agreed that the practice of sharing notes had changed their charting, 25.7% (83/323) stated that they now consider their notes to be less clinically valuable, 72.4% (234/323) said that there had been no change in value, and 1.9% (6/323) stated that they believe that their notes are more valuable. Of the 86 participants who believe that their clinician notes are now less helpful to other healthcare professionals, 83 (97%) documented that they have experienced a change in how they chart. A total of 519 participants answered that there was no change in chart value, with 234 (45.1%) stating that they have changed the way they chart and 285 (54.9%) responding that they have not altered their charting.

**Table 4:** Polychoric correlations.^a^

Variables	Opinion of open notes	Hospital department	Hospital position	Useful to engage patient	Speak with patient before they access notes	Chart time	Changed my charting	Clinical value
Opinion of open notes^b^	—^c^	—	—	—	—	—	—	—
Hospital department	–0.01	—	—	—	—	—	—	—
Hospital position	0.13	–0.02	—	—	—	—	—	—
Useful to engage patient^b^	0.83	0.01	0.10	—	—	—	—	—
Speak with patient before they access notes (no=0, yes=1)	0.16	0.16	–0.06	0.11	—	—	—	—
Charting time^d^	–0.13	–0.11	0.33	–0.08	–0.21	—	—	—
Changed my charting (no=0, yes=1)	0.40	0.01	–0.08	0.29	0.29	–0.51	—	—
Clinical value^e^	0.48	0.08	–0.21	0.42	0.28	–0.25	0.54	—
Encourage reading (yes=1, no=2)	0.48	–0.06	0.07	0.51	–0.19	–0.07	0.11	0.36

^a^Computed by the R Hector package in SPSS software (version 26.0; IBM Corp).

^b^Response options: 1=“Agree,” 2=“Somewhat agree,” 3=“Neutral,” 4=“Somewhat disagree,” and 5=“Disagree.”

^c^Not applicable.

^d^Response options: 1=“More time writing notes,” 2=“No change,” and 3=“Less time writing notes.”

^e^Response options: 1=“More valuable,” 2=“No change,” and 3=“Less valuable.”

## Discussion

### Principal Findings and Comparison With Prior Work

The 21st Century Cures Act has revolutionized the way patients interact with the healthcare system and has produced novel changes in practice for clinicians. Past research on the effects of sharing visit notes has focused mainly on the patient experience [3-9], with little investigation into its impact on clinicians. In addition, these previous studies were conducted within institutions that willingly adopted the practice and were limited in the diversity of clinician participants and study time periods. The primary objective of this study was to assess clinicians’ initial experiences with, and attitudes toward, sharing visit notes with patients after being mandated to do so by the Cures Act. The secondary objective was to determine clinician preferences regarding instant record release. This study was conducted soon after the implementation of the Final Rule mandate to identify areas for improvement and the potential need for intervention during this transition period.

Similar to previous studies [17-19], most of the clinicians (355/613, 57.9%), aside from registered nurses, considered open notes to be a good idea. Clinicians who disagreed (or were neutral) were more likely to experience changes in their charting and increased charting time. These effects may likely have negatively affected their opinion of the practice. Although most of the clinicians (340/613, 55.5%) view shared notes as useful tools for engaging patients, the majority (470/612, 76.8%) do not encourage their patients to read their notes; therefore, the previously documented patient benefits may not be as pronounced. These associations demonstrate a need for clinician intervention. A recent study examined the impact an open notes educational course had on clinicians [23]. Multiple benefits were documented, including an improved ability to use shared notes as a tool for patient engagement, increased clinician encouragement of patients to view their notes, and decreased worry of the practice’s negative consequences. A similar intervention may benefit the clinician population experiencing adverse effects after the Cures Act. The differing opinion of registered nurses on the value of shared notes reflects a need for a more in-depth investigation into how sharing clinical notes affects each discrete health profession discipline. In addition, future studies should assess whether different specialties have different areas of concern regarding the changes in practice mandated by the Cures Act, such as the instantaneous release of records versus sharing the content of clinician notes.

Most of the clinicians (323/612, 52.8%) noted a change in their clinical documentation, an effect that is also reflected in the literature [15-17]. Clinicians most often reported changes in their use of language that could be perceived as critical to the patient and how they document sensitive information. In an open-ended portion of the survey, multiple respondents reported their concern that sharing notes with patients has negatively affected communication among health professionals, a worry that other clinicians have previously reported [15]. Comments that notes are now written more with the patient in mind than other health professionals may explain this finding. In addition, although most of the clinicians (323/612, 52.8%) reported a change in their charting without an immediate alteration in the clinical value of their note, the long-term effects of the reported changes in medical documentation should be examined. Exclusion of information from clinical notes may potentially affect the efficacy and efficiency of care in the long term. Withholding information may make it more challenging to share diagnostic and treatment processes with consulting clinicians, leading to wasted time and resources. In addition, the noted omission of specific differential diagnoses from patient records may affect treatment or diagnostic processes later in the patient’s care. Future studies should assess clinician opinion of the utility and clinical value of other health professionals’ notes after the Cures Act because this may be a better measure of the true value of the note that is meant for interprofessional communication and continuity of care.

The impact that open notes have had on the privacy of patients’ protected health information was not directly assessed in this study; however, a clinician reported concern surrounding increased access to notes by individuals other than the patient, particularly as it pertained to adolescents and those experiencing domestic violence. This worry is one that has been echoed by others in the medical community [24-26]. Although the protection of patient privacy was not the focus of the study, one must recognize that unauthorized access to patients’ private health information is problematic. Future studies should explore measures to decrease and prevent this threat to patient privacy.

Most of the clinicians (431/613, 70.6%) agreed that it is essential to speak with patients before their records are released. When provided the opportunity to discuss their experiences further, some of the clinicians (13/168, 8%) documented that the automatic release of records has adversely influenced their practice and well-being. A clinician spoke about a newfound sense of burnout owing to an increase in the number of telephone calls and messages they receive from patients who view their records before the clinician can review reports with them. This consequence of instantaneous record release has also been demonstrated elsewhere. According to a recent study conducted by Steitz et al [27], the median number of daily messages sent by patients within 6 hours of reviewing a test result released without any delay doubled after the Cures Act. In addition, multiple clinicians noted that the instant release of pathology reports has resulted in a loss of the ability to thoughtfully deliver bad news. In addition to the negative effects of instant record release reported by clinicians, it has previously been demonstrated that open notes have the potential to disrupt the physician-patient relationship [16]. Analysis of responses to this survey exhibited this effect, with some of the clinicians (6/168, 4%) reporting increased patient distrust of healthcare professionals. The effect of shared notes on the clinician-patient relationship and the convention of the automatic record release should be assessed in subsequent studies. These findings may illustrate an area for improvement in the current Cures Act protocol.

Before the Cures Act, the University of Kansas Health System had yet to provide patients with digital access to clinician notes. This policy regulation and that of the instant release of patient records were novel changes in practice for the participating clinicians. Leading up to the deadline for Cures Act compliance, the health system implemented a 6-month timeline of graduated physician education and awareness. Physician informaticists and physician executives accomplished this through a series of townhall, chair, and department administrator meetings.

The findings of this study may be generalizable to institutions similar to the University of Kansas Health System, and the clinician testimonies gathered in this study may provide valuable insight into the initial opinions and experiences of clinicians at these institutions. In addition, these clinician experiences collected early in the transition period may be used to guide future health policy implementation and understand how best to prepare clinicians for these changes in practice.

### Limitations

This study includes multiple limitations. First, because the survey was conducted soon after the mandated changes in practice went into effect, clinicians with opinions and experiences more extreme than those of nonrespondents may have been more likely to participate, resulting in self-selection bias. Second, clinician knowledge of past study findings reporting positive patient benefits from sharing visit notes may have introduced response bias because some clinicians may have had a preexisting positive opinion of the practice. In addition, the research team failed to ask participants whether they were aware that patients were reading their notes, thereby increasing the potential for response bias. In addition to the long-standing issue of poor clinician participation rates in survey studies [28-30], the modest response rates may be attributed, in part, to the concurrent COVID-19 pandemic and the considerable constraints that study participants were experiencing at the time of data collection. Differences in participant recruitment methods may have also had some bearing on the response rates and acquired data, particularly for the resident physician population because graduate medical education policies prohibited direct solicitation for participation. Alternatively, a link to the survey was posted on the institutional resident website, potentially limiting the recruitment of this target population because many may not have seen the invitation to participate. In addition, the national nursing shortage may have contributed to the suboptimal response rate of nurses.

### Conclusions

The Cures Act requires health systems to provide patients with free, uninhibited digital access to their medical records. This new legislation is a prime example of the new patient-centered approach the US healthcare system has taken toward care. Previous studies have documented the perceived benefits shared visit notes have on the patient experience; however, the effect on clinicians has not been as widely studied.

Although the Cures Act has revolutionized the role that patients are able to have in their health care, it has also brought about novel changes in clinical practice, notably shared visit notes and the instant release of records. The impact these changes in practice have on clinicians of all disciplines and specialties should be further assessed in future studies. Educational interventions for health professionals and patients may alleviate some of the adverse effects that clinicians are experiencing with this transition in practice and maximize potential benefits. In addition, it is essential to monitor the effect that both shared notes and instant record release have on the clinician-patient relationship and clinicians’ charting mores.

## Appendix

Multimedia Appendix 1 Cures Act Clinician Survey 2021.

Multimedia Appendix 2 Additional clinician testimony.

## Abbreviations

ICU: intensive care unit
REDCap: Research Electronic Data Capture
