# Supplementary material for: Attitudes and Experiences of Clinicians After Mandated Implementation of Open Notes by the 21st Century Cures Act: Survey Study
Source: J Med Internet Res. 2023 Feb 28;25:e42021. doi: 10.2196/42021 (PMC10015345; doi:10.2196/42021)
Supplement: Multimedia Appendix 1 [file jmir_v25i1e42021_app1.docx]

Appendix: OpenNotes Clinician Survey 2021

1. (hospital_department)

What department of the University of Kansas Hospital do you work in?

- Anesthesiology
- Cardiovascular Medicine
- Emergency Medicine
- Family Medicine
- General Surgery
- Internal Medicine
- Neurology
- Neurosurgery
- Obstetrics and Gynecology
- Ophthalmology
- Orthopedic Surgery
- Otolaryngology and Head and Neck Surgery
- Pathology and Laboratory Medicine
- Pediatrics
- Plastic Surgery
- Psychiatry and Behavioral Sciences
- Radiation Oncology
- Radiology
- Rehabilitation Medicine
- Urological Surgery
- Other

1. (hospital_position)

What is your position within the department?

- Attending Physician
- Resident Physician
- Advanced Practice Provider
- Registered Nurse

1. (visit_notes)

How much do you agree that making visit notes available is a good idea?

- Agree
- Somewhat agree
- Neutral
- Somewhat disagree
- Disagree

1. (useful_tool)

How much do you agree that open notes are a useful tool for engaging patients in their care?

- Agree
- Somewhat agree
- Neutral
- Somewhat disagree
- Disagree

1. (patient_conversation)

Is it important to you that you speak to your patients about certain records prior to them accessing the records?

- Yes 🡺 delayed_access
- No

1. (delayed_access)

If you answered yes to the question, "Is it important to you that you speak to your patients about certain records prior to them accessing the records?", which kinds of records do you wish to be delayed access? (select all that apply)

- Labs
- Radiology
- Other 🡺 delayed_access_other

1. (delayed_access_other)

Please describe.

1. (charting_time)

Because of open notes do you spend

- More time writing notes
- No change
- Less time writing notes

1. (opennotes_change)

Do you believe open notes has changed the way you chart?

- Yes 🡺 opennotes_explanation
- No

1. (opennotes_explanation)

If you answered yes to the question, "Do you believe open notes has changed the way you chart?", mark what type of change (if applicable):

- Use of language that could be perceived as critical to the patient
- How you document sensitive clinical, mental health, or social information
- How you document patients' perspectives, preferences, and concerns
- Use of terms such as "noncompliant", "patient refuses", and "patient denies"
- Use of medical jargon or abbreviations
- Other 🡺 visit_note_additional

1. (visit_note_additional)

Please comment on any additional changes.

1. (note_value)

Has open notes affected the clinical value of your notes for other clinicians?

- More valuable 🡺 value_explanation
- No change
- Less valuable 🡺 value_explanation

1. (value_explanation)

Please explain.

1. (notes_encouragement)

Do you encourage your patients to read their notes?

- Yes
- No

1. (additional_comments)

If you have any additional comments you would like to make about your experience with open notes, please describe them below.
