# Supplementary material for: Attitudes and Experiences of Clinicians After Mandated Implementation of Open Notes by the 21st Century Cures Act: Survey Study
Source: J Med Internet Res. 2023 Feb 28;25:e42021. doi: 10.2196/42021 (PMC10015345; doi:10.2196/42021)
Supplement: Multimedia Appendix 2 [file jmir_v25i1e42021_app2.docx]

Table. Supplementary Table of Quotes

| **Theme** | **Variable** | **Quote** |
| --- | --- | --- |
| If you answered yes to the question, "Is it important to you that you speak to your patients about certain records prior to them accessing the records?", which kinds of records do you wish to be delayed access?  If you answered yes to the question, "Is it important to you that you speak to your patients about certain records prior to them accessing the records?", which kinds of records do you wish to be delayed access? | delayed_access_other  delayed_access_other | “All data in our notes can be misconstrued if they are read prior to discussing the plan for the day. I work in the ICU, reading the daily notes has caused our patients and families more anxiety than being helpful.” |
|  |  | “Radiation oncology treatment data (daily treatment data) are available to patients. They have a great deal of difficulty figuring out what this information means, it is confusing, they often think it does not align with what 'the internet' implies their total treatment dose should be, and that causes concern.” |
|  |  | “Pathology results are anxiety provoking and require interpretation. CT imaging has a lot of medical language that can cause anxiety for pts when read without provider interpretation.” |
|  |  | “While I'm not a clinician/don't see patients directly, I do think it's very important for clinicians to have the opportunity to review surgical pathology results with the patient before the patient views the results. As a surgical pathologist I often report out new cancer diagnoses, and sometimes the findings of the cancer are unexpected. I think it is in the patient's best interest to receive news of a new cancer diagnosis from their provider, who can also give an idea of prognosis or next steps, rather than finding the information on their own without any support or access to other information.” |
|  |  | “Pathology results - patients may find out they have cancer before the ordering physician has had a chance to review pathology. Alternatively, the pathology may be completely benign, but the patient may believe they have cancer (adenoma vs adenocarcinoma) and this may lead to unnecessary patient stress and anxiety.” |
|  |  | “Part of being a physician is interpretation of results in the context of each patients’ issues. This is being taken away from our practice and will have profound negative impact on the PP relationship.” |
|  |  | “All encounters are subject to gross misinterpretation by medically naive persons. When we write for the lay public, we're told to target audience with less than high school education. The idea that patients could gain peace of mind or insight about their health from reading material created by and for medical professionals with advanced training is untested and seems implausible.” |
|  |  | “I have had patients get upset because of functional diagnoses, substance use diagnoses, and personality disorders.” |
|  |  | “I prefer to review labs and radiology reports prior to the patient so that if worsened data is present, I can explain the results in a less alarming manner to the individual who is often ill.” |
|  |  | “When a patient reads the formal report, it has words many patients do not understand. Some will turn to Dr. Google and try to figure out what is going on. Dr. Google takes them down a rabbit hole, and they are often very scared unnecessarily.” |
|  |  | “As a surgeon, I am not constantly sitting in front of the EMR during my work hours. I also make every effort to avoid EMR after hours to improve my own quality of life. This situation simply adds to physician burnout.” |
|  |  | “I treat cancer patients and a lot of results can be scary without understanding what we are looking for. Radiology and pathology results have a lot of language that is hard to understand, and patients become very stressed about these until they can talk to a provider. I used to call with results or discuss at visits, now they get results sooner and call us in a panic.” |
|  |  | “Often times, patients and their families are accessing and reviewing labs, imaging reports and consultant notes prior to having spoken to a physician about those results. Clinicians are absolutely necessary to provide context and meaning to results and more importantly, to prepare patients to receive "bad news" such as a diagnosis of malignancy. This has proven difficult on inpatient medicine services, when physicians are confronted with a multitude of questions about irrelevant labs or insignificant findings/incidental findings on radiology reports.” |
| Do you believe open notes has changed the way you chart? | visit_note_additional | “Must respond to more patient questions before appt, requests to change documentation. Must act immediately in results as patients further expect immediate interpretations and communication.” |
|  |  | “Careful parsing of language to avoid legal pitfalls.” |
|  |  | “I am a palliative care physician, so I have become more discrete and less objective in my documentation of family meetings and psychosocial issues.” |
|  |  | “Can no longer comment on sensitive maternal health issues / concerns for domestic violence etc, relevant to the overall plan for the child, in the chart as partner would also have access to the child's notes. This makes interdisciplinary communication prone to missing vital information.” |
|  |  | “Information I want to convey to another physician about diagnosis or treatment takes much more time to rephrase in a manner a patient understands.” |
|  |  | “I had already stopped saying things like "non-compliant," and "patient refuses." This just supports that move and helps us show respect for our patients in that way. I don't omit much in terms of sensitive topics, but I do double-check to make sure I have captured the patient's issues accurately.” |
|  |  | “I very much hesitate to document patient abusive behavior and drug seeking.” |
| Has open notes affected the clinical value of your notes for other clinicians?  Has open notes affected the clinical value of your notes for other clinicians?  Has open notes affected the clinical value of your notes for other clinicians? | value_explanation  value_explanation  value_explanation | “The notes are less valuable in general. Censoring the note means that valuable information may be excluded. Ex. "Obesity" is an objective measurement determined by height and weight. Obesity impacts LOTS of secondary health issues. "Obesity" makes patients feel bad and they call to have it removed from their note.” |
|  |  | “When clinical commentary is altered to be more easily interpreted by lay people, it adds words, reduces efficiency and makes it harder for other physicians to wade through unnecessary material to find needed information.” |
|  |  | “Notes are no longer meaningful. They simply document a few facts but have no thought process or differential diagnosis thoughts.” |
|  |  | “Feel "watched" and untrusted.” |
|  |  | “Harder to document when patients are unstable and not a good candidate for surgery. Have to 'dance' around sensitive issues.” |
|  |  | “Often things are omitted from notes now because of patient's access. Pertinent social and other history is important for other providers to see. Now that patients have access to the notes, they may perceive this as negative, and providers are being harassed because of such documentation.” |
|  |  | “Sometimes I have some concerns about the validity of the evaluation or some emotional overlay in the symptoms. I no longer feel comfortable noting that in my chart. This is likely to give other clinicians a false impression.” |
|  |  | “Documenting patient prognostic awareness is more challenging, so now clinicians who read my palliative care notes will know less about patient prognostic awareness.” |
|  |  | “They do not need the added discussion and language I now find myself adding to notes for the benefit of only the patient. It adds to already voluminous amount of documentation.” |
|  |  | “In some instances, I feel like I am not being truthful in explaining a situation due to a patient being able to read about my concern. Also, documentation on minors when a parent might have access to their chart, hard to be truthful about conversations that were had in confidence.” |
|  |  | “Providers are trying to be more delicate in the way they word things and therefore it can be difficult to discern nuances.” |
|  |  | “It's challenging to document the true noncompliance of a patient and escalation of things to MDs when the patient does not agree with the assessment. It puts the RN in a difficult position.” |
|  |  | “I believe initially notes were for the physician. Then, notes became a tool for the insurance companies as well as the physician. This third stage it appears the notes are no longer really for the physician, but for the patient.” |
|  |  | “If for the patients’ safety I do not chart certain sensitive clinical, mental or social information, I do not have a way to get that information back to the patients primary care provider for continuity of care.” |
|  |  | “I have spent my career making my notes useful to other medical personnel, including nurses and other doctors in my practice or consulting physicians. It has been important in the past for others to understand my thought process and why I might be seeking a certain route of evaluation or treatment. I now feel that I need to write my note with wording that is understandable to someone with no medical knowledge and that does not elicit anxiety or fears. I give more thought and time to being careful about how I share my thoughts/plan so as not to cause undue stress for the patient.” |
|  |  | “I believe with open notes the physicians, ancillary staff and others involved in patient care helps to incorporate a more thorough involvement in those in the care of the patient.” |
|  |  | “If I can't use my training to quickly convey information using jargon and abbreviations, and I don't have time to translate medical terms for patients, I'm not able to provide as much info as I used to provide.” |
|  |  | “As I read other clinician's notes, there is less clinical narrative in assessment and plan.” |
|  |  | “Increased self-censorship of my notes has resulted in less accurate language used and often times omitted due to fear of causing dissatisfaction for patients. Physical exam components regarding body habitus also not included as often (despite medical accuracy) for concerns of being considered offensive by patients. Mental status and psychiatric documentation also less descriptive now for reasons stated above.” |
